# Overcoming difficulties with equipoise to enable recruitment to a randomised controlled trial of partial ablation vs radical prostatectomy for unilateral localised prostate cancer

**DOI:** 10.1111/bju.14432

**Published:** 2018-08-15

**Authors:** Daisy Elliott, Freddie C. Hamdy, Tom A. Leslie, Derek Rosario, Tim Dudderidge, Richard Hindley, Mark Emberton, Simon Brewster, Prasanna Sooriakumaran, James W.F. Catto, Amr Emara, Hashim Ahmed, Paul Whybrow, Steffi le Conte, Jenny L. Donovan

**Affiliations:** ^1^ Population Health Sciences Bristol Medical School University of Bristol Bristol UK; ^2^ NIHR Collaboration for Leadership in Applied Health Research and Care West at University Hospitals Bristol NHS Trust Bristol UK; ^3^ Nuffield Department of Surgical Sciences University of Oxford Oxford UK; ^4^ Department of Oncology and Metabolism University of Sheffield Sheffield UK; ^5^ University Hospital Southampton NHS Foundation Trust Southampton UK; ^6^ Hampshire Hospitals NHS Foundation Trust Basingstoke UK; ^7^ Division of Surgery and Interventional Science University College London London UK; ^8^ University College London Hospital NHS Foundation Trust London UK; ^9^ Imperial Urology Imperial College Healthcare NHS Trust London UK

**Keywords:** equipoise, feasibility, qualitative, recruitment, randomised controlled trial, #PCSM, #ProstateCancer

## Abstract

**Objective:**

To describe how clinicians conceptualised equipoise in the PART (Partial prostate Ablation vs Radical prosTatectomy in intermediate‐risk unilateral clinically localised prostate cancer) feasibility study and how this affected recruitment.

**Subjects and Methods:**

PART included a QuinteT Recruitment Intervention (QRI) to optimise recruitment. Phase I aimed to understand recruitment, and included: scrutinising recruitment data, interviewing the trial management group and recruiters (*n* = 13), and audio‐recording recruitment consultations (*n* = 64). Data were analysed using qualitative content and thematic analysis methods. In Phase II, strategies to improve recruitment were developed and delivered.

**Results:**

Initially many recruiters found it difficult to maintain a position of equipoise and held preconceptions about which treatment was best for particular patients. They did not feel comfortable about approaching all eligible patients, and when the study was discussed, biases were conveyed through the use of terminology, poorly balanced information, and direct treatment recommendations. Individual and group feedback led to presentations to patients becoming clearer and enabled recruiters to reconsider their sense of equipoise. Although the precise impact of the QRI alone cannot be determined, recruitment increased (from a mean [range] of 1.4 [0–4] to 4.5 [0–12] patients/month) and the feasibility study reached its recruitment target.

**Conclusion:**

Although clinicians find it challenging to recruit patients to a trial comparing different contemporary treatments for prostate cancer, training and support can enable recruiters to become more comfortable with conveying equipoise and providing clearer information to patients.

## Introduction

Patients with intermediate‐risk localised prostate cancer are usually offered radical prostatectomy (RP) or external beam radiotherapy with a view to curing the cancer, although these can result in substantial urinary, bowel, and sexual function side‐effects 1. Partial ablation (PA) techniques have been developed to target the cancer, preserving the rest of the prostate, and thus aiming to reduce treatment side‐effects. These techniques include high‐intensity focused ultrasound (HIFU), cryotherapy, photodynamic therapy, brachytherapy, and radiofrequency interstitial tissue ablation. A systematic review reported that PA rarely causes significant morbidity and appears to have a reduced impact on quality of life, although findings are based on a few experienced centres and data from non‐randomised studies on oncological effectiveness or impact on functional outcomes and quality of life 2.

The PART (Partial prostate Ablation vs Radical prostaTectomy; ISRCTN 99760303) feasibility study aimed to recruit 80 men with intermediate‐risk unilateral clinically localised prostate cancer (defined as Gleason grade score 7 (3+4 or 4+3), >4 mm cancer core length, PSA level ≥20 ng/mL, clinical ≥T2b disease) to a randomised controlled trial (RCT) comparing PA or RP. HIFU was identified as the most promising PA technology at the time of trial development 3. However, delivering an RCT of PA against RP was anticipated to be particularly challenging because of the likelihood of strong views amongst clinicians and patients about two very different treatments 4.

Given that recruitment was anticipated to be difficult, an embedded QuinteT Recruitment Intervention (QRI) aimed to understand, and subsequently optimise, recruitment 5. The QRI was developed initially for the Prostate Testing for Cancer and Treatment (ProtecT) trial and has been implemented subsequently in 25 RCTs 6. The QRI identified several issues that affected recruitment in PART, including organisational barriers and recruiter difficulties with explaining the trial to potential patients 7. The present article focuses on how clinicians conceptualised equipoise in PART, how this changed during the QRI and in their presentations of information to patients, and what lessons could be learned for future trials.

## Subjects and Methods

### Study Design

The QRI involved two iterative phases: Phase I, which sought to identify and understand recruitment difficulties (through analysis of screening logs, interviews with trial staff, and audio‐recording consultations where PART was discussed with patients); and Phase II, which implemented strategies to optimise recruitment and informed consent. The study is reported according to qualitative reporting guidelines (Table S1). Ethics approval was provided by the NHS Health Research Authority National Research Ethics Service (NRES) Committee London – Camden and Kings Cross (14/LO/0640). Written informed consent was provided by all participants.

### Data Collection

Data were collected in three ways:

#### Interviews

Semi‐structured interviews were conducted with members of the Trial Management Group (TMG) and healthcare professionals who were involved in recruitment. Separate topic guides were developed for the TMG and recruiters (Appendix S1) to ensure coverage of overall study issues (TMG) and recruitment (recruiters), with sufficient flexibility to allow for new issues to emerge. Interviews were transcribed verbatim, checked against the audio‐recording for accuracy, and transcripts were imported into NVivo (version 10, QSR International).

Data were analysed by D.E. using techniques of constant comparison derived from grounded methodology, which aims to generate new theories about phenomena that develop from, or are ‘grounded’ in, the data as they are collected and compared with existing findings to identify similarities and differences 8. Emerging themes were discussed with J.L.D. with reference to the raw data. Equipoise was considered to be present if recruiters conveyed that patients would not be advantaged or disadvantaged if they were to receive either procedure (as far as existing evidence would dictate) 9. Any instances where clinicians described certainty or uncertainty around treatment superiority, or any discussion/practices that suggested that treatments were equivalent or one would be better or worse for the patient were coded.

#### Recorded Recruitment Consultations

Healthcare professionals recruiting to PART were requested to audio‐record appointments where they provided information to eligible patients about the study and treatment options. Recordings were transcribed verbatim, and for this analysis, selected parts related to equipoise issues were extracted. These were analysed as described above for interviews, with the addition of some of the techniques of focused conversation analysis to identify and document aspects of informed consent and information provision that was unclear, disrupted or hindered recruitment 5.

#### Patient Pathway Through Eligibility and Recruitment

Screening logs from all centres were examined regularly for information on the number of patients screened, eligible, approached, and randomised 10, to provide contextual information about recruitment in clinical centres and across the study.

## Results

### Interviews

The QRI team approached 23 participants to take part (including members of the TMG, and representatives from each recruiting site). A total of 13 one‐to‐one interviews were conducted between July and November 2015 by two researchers (D.E. and P.W.). The final sample included 12 recruiters (four of whom were members of the TMG) and one non‐recruiting TMG member. Interviews lasted a mean (range) of 43 (31–53) min.

### Recorded Consultations

In all, 64 recruitment appointments with 54 patients were audio‐recorded (ten patients had two consultations recorded) between September 2015 and April 2017: 24 as part of QRI Phase I and 40 after feedback in Phase II. Consultations lasted a mean (range) of 27 (10–42) min. In all, 12 different recruiters led the consultations. Audio‐recordings were obtained from four recruiting sites (Table 1).

**Table 1:** Overview of data collected from centres

Site	Number of recruiters	Number with previous training	Number interviewed	Number of recordings before feedback	Overview of feedback	Number of recordings after feedback
Centre 1	5	1	5	9	Group feedback (×3), tips document, individual feedback to one recruiter	3
Centre 2	3	0	2	2	Group feedback, tips document	0
Centre 3	3	0	2	6	Group feedback (×3), tips document, individual feedback to one recruiter	17
Centre 4	3	0	1	7	Group feedback (×2), tips document, individual feedback to one individual (twice)	20
Centre 5	3	3	2	0	Group feedback (×2), tips document	0

Quotations are provided to support the results, and distinctions are made between data from interviews and recorded consultations. Quotes were anonymised to ensure confidentiality.

### Phase I: Understanding Recruitment Challenges

#### Views on the Study Design

In interviews, there was clear enthusiasm for the PART study, and HIFU was described as an ‘exciting’ and ‘promising’ alternative to radical procedures. Some urologists commented that comparing only one form of PA vs one radical treatment (surgery) might exclude patients who expressed preferences for treatment options outside PART (such as radiotherapy or brachytherapy). However, there was consensus that more data were available for HIFU. The ProtecT trial had not yet been published and so RP was deemed the most appropriate comparator.

Interview, Recruiter 1:‘I think this trial is needed because there has been a lot of hype or buzz about HIFU and focal therapy for some years now.’

#### Previous Recruitment Experience

Recruitment to RCTs was acknowledged to be challenging, particularly discussing concepts such as uncertainty and randomisation. Many participating clinicians had not received training for their role as recruiters, with the exception of four recruiters who had participated in the ProtecT study and appeared more comfortable with the concept of randomisation and how to convey uncertainty to potential trial patients:

Interview, Recruiter 2: ‘I haven't, personally, been responsible for recruiting to trials […] I have no idea whether HIFU's going to work or not, so it makes it very difficult to know how much of that information to tell patients.’Interview, Recruiter 15: ‘I think we need to be confident on our uncertainty, and you know, I've learned a lot by being involved in ProtecT […] We acknowledge that there are uncertainties in the decision‐making, which is why we run clinical trials.’

#### Discomfort with the Eligibility Criteria

Recruiters often described that they felt that some patients, although fulfilling the study's eligibility criteria, were more suitable for a particular treatment option. This meant that not all eligible patients were necessarily invited to be enrolled in PART. When patients were approached, the question of whether they were eligible for participation affected how clinicians communicated with patients. Examples of this are shown in Table 2.

**Table 2:** Recruiter perceptions of the PART eligibility criteria

Inclusion criterion, according to protocol	Examples of recruiter discomfort
Gleason grade score 7 (3+4 or 4+3)	Interview, Recruiter 12: ‘I was just marginally uncomfortable because he had a 4+3 and he was 50 years old and it was quite a significant volume of tumour. I just found myself thinking, “Do you know what? I wonder if you'd be better off having a radical prostatectomy.”’ Interview, Recruiter 9: ‘3+4's, I think they are maybe the best ones to treat with HIFU, where they've got mainly pattern 3, but a bit of pattern 4. I think you can be pretty confident that you're going to wipe out that pattern 4 when you do the treatment.’
High volume Gleason grade score 6 (>4 mm cancer core length)	Interview, Recruiter 15: ‘If somebody had all of the cores from one side, let's say every single core from one side was involved from a mapping biopsy and it was involved with like 80% of four plus three but the other side is completely clear. In theory he is a PART candidate. But actually maybe he is better off with a prostatectomy.’
Life expectancy ≥10 years	Interview, Recruiter 9: ‘I think for some patients let's just say in their 70s, let's say between 70 and 75, I have absolutely no problem saying, “Surgery or radiotherapy, it doesn't matter which one you have. Just choose the one for which the conduct of therapy and the side effects feels best to you”. For the people perhaps between 65 and 70 I'm sort of in the same opinion but perhaps slightly leaning towards surgery. For the under 65s I really think that surgery is probably better because of the life expectancy they probably have and the risk of failure going into the long‐term and the long‐term burden of even fairly mild toxicity from radiotherapy.’

#### Recruiter Bias

In interviews, those who had not received support or training for their role as recruiters sometimes expressed strong preferences for a particular treatment. Advocates of RP expressed concerns that HIFU would not remove all of the cancer, whereas those who favoured HIFU expressed concerns that surgery would be over‐treating cancer and compromising quality of life unnecessarily. Consequently, recruiters found it difficult to express equipoise (Table 3).

**Table 3:** Recruiter perceptions of PART treatment options

Recruiter	Examples of recruiter bias
Recruiter 6	‘There's very few patients with whom I still have equipoise with as to whether they should have HIFU or prostatectomy.’
Recruiter 9	‘It's just whether that a prostatectomy is over treating their cancer… and I've got to be honest with them.’
Recruiter 1	‘They are compromising their cancer treatment by taking the risks that we're only treating one part of the prostate. And so there might be another part of the prostate which has some prostate cancer in. So that they understand that, after I've told them.’
Recruiter 12	‘I think of the patients who have been suitable for both surgery and HIFU I have to say I probably steered them towards HIFU whenever they have been suitable.’

Consultations showed that these beliefs were conveyed to patients. There were several instances where the concept of uncertainty was not introduced and biased terminology was used, such as ?gold standard’ RP and ?experimental’ HIFU. Recruiters also provided unbalanced accounts of the procedures (e.g. discussing primarily the advantages of HIFU and the disadvantages of RP). Sometimes direct treatment recommendations were provided. Following this, patients tended to express clear preferences for a specific treatment and declined participation in PART.

Consultation, Recruiter 2: ‘If you have surgery, with the kind of disease you have, you'd almost certainly not die of prostate cancer.’ (Patient declines PART, opts for RP)Consultation, Recruiter 9: ‘You've then the option of partially destroying the part of the prostate where the cancer is, and in your case, it's on the left‐hand side. That's called ‘focal destruction’. […] It's all done very cleverly […] It is a potentially attractive option […] It's quite favourable.’ (Patient declines PART, opts for HIFU)

Between January and November 2015 (during the first phase of the QRI), 15 men had been recruited and the mean (range) number of patients agreeing to be randomised was 1.4 (0–4) patients/month, with a conversion rate (the numbers of eligible men invited to join PART who then went on to be randomised) of 20% (15/75). The lead site had recruited most of these, whilst some sites had not recruited any patients in that time (Table 4).

**Table 4:** Recruitment by each centre

Centre	2015				2016				2017
	January–March	April–June	July–September	October–December	January–March	April–June	July–September	October–December	January–March
Site 1	4	5	2	4	4	2	5	2	9
Site 2	*Activated 05/15*								
Site 3	*Activated 06/15*			1	2	5	1		1
Site 4	*Activated 10/15*			1		2		3	4
Site 5	*Activated 11/15*			2		7	4	2	10
Total	4	5	2	8	6	16	10	7	24

### Optimising Recruitment

#### Summary of Training

In November 2015, the QRI team presented the findings of Phase I to the Chief Investigator and TMG, and strategies to improve recruitment and informed consent were developed (Table 5). These included group feedback, individual feedback, and the production of ‘tips’ documents (Appendix S2). Group sessions were interactive, with open discussions encouraged. Overall QRI feedback and training focussed on:

**Table 5:** Summary of PART recruitment interventions

Date	PART recruitment interventions
November 2015	Preliminary QRI findings discussed with Chief Investigator
December 2015	Full descriptive report on recruitment issues sent to Chief Investigator
December 2015	Two‐part recruitment session at Collaborator's meeting
December 2015	Group feedback session at Centre 3
December 2015	Recruitment e‐mail sent to all recruiters
December 2015	Recruitment newsletter sent to all recruiters
February 2016	Tips document sent to each recruiter
February 2016	Recruitment newsletter sent to all recruiters
February 2016	Individual feedback meeting with recruiter from Centre 1
March 2016	Group meeting at Centre 2 to discuss recruitment
March 2016	Group feedback session at Centre 3
March 2016	Website updated to include patient information about PART
April 2016	Recruitment newsletter sent to all recruiters
April 2016	Group feedback session at Centre 1
April 2016	Group feedback session at Centre 5
May 2016	Recruitment newsletter sent to all recruiters
May 2016	Individual feedback meeting with recruiter from Centre 4
May 2016	Individual feedback meeting with recruiter from Centre 3
*Funding variation – July 2016–September 2016*	
September 2016	Recruitment newsletter sent to all recruiters
October 2016	Recruitment e‐mail sent to all recruiters
October 2016	Group feedback to Centre 3
January 2017	Recruitment e‐mail to all recruiters
January 2017	Individual feedback with recruiter from Centre 3
January 2017	Group feedback session at Centre 1
February 2017	Recruitment e‐mail sent to all recruiters
March 2017	Individual feedback with recruiter from Centre 4

- Ways in which recruiting to RCTs differs to standard practice.
- The lack of randomised evidence comparing RP with HIFU.
- The extent to which there was community equipoise (i.e. by demonstrating the conflicting biases for the treatment arms).
- Examples of how recruiter beliefs could influence patient preferences.
- The importance of exploring preferences to ensure men were making a fully informed decision.

#### Changes to Recruitment and Informed Consent

Phase II of the QRI began in November 2015, and continued for the remainder of recruitment. During this time, a mean (range) of 4.5 (0–12) patients/month were randomised and the conversion rate increased from 20% (15/75) to 42% (67/161). Furthermore, after the initial intervention in December, several centres (rather than predominately Site 1) began recruiting consistently (Table 3). Analysis of the recordings available after feedback highlighted changes in the ways recruiters discussed the study and treatment options (Table 6).

**Table 6:** Before and after feedback

Recruiter	Before feedback	After feedback
	Consultation: Recruiter: ‘I think you are most suitable for focal treatment with HIFU […] I think the research study that I was going to be talking about is probably not relevant for you.’	Consultation: Patient: ‘What do you think?’ Recruiter: ‘I've been a consultant for years and I sit here telling men what's going on, telling them they've got cancer, they ask me what treatment we go for, and I try and help them and steer them in the direction, but, at the end of the day, I have to sometimes stop and say, “Actually, there isn't really any evidence that this treatment is better than that one because there have never been any proper trials comparing properly treatment A with treatment B. To get proper results, you have to actually do it in a randomised way.” There is a study called PART, that we're very much involved with…’
Recruiter 9	Consultation: Patient: ‘What would be your advice? Which treatment, in this particular case?’ Recruiter: ‘I think that surgery or radiotherapy, for someone who is young and fit like you, with slightly more bulky disease, would be more appropriate. So, I don't think the trial is right for you.’	Consultation: Recruiter: ‘I don't think that there's an obvious, “You must go one way or the other.” The reality is, there's such a lack of evidence, we just don't know. I'm a fan of both treatments, if that makes sense, and regularly I'm referring people for both sorts of treatments […] The more I have these discussions and the more I reflect on it all, it does make me think, “You know what [name] a lot of what you say is based on very little evidence.” We don't really know whether treatment A is better than treatment B.’
Recruiter 12	Interview: Recruiter: ‘I think of the patients who have been suitable for both surgery and HIFU I have to say I probably steered them towards HIFU whenever they have been suitable. Partly I am trying to build my experience and partly it is a less toxic treatment. It fits in with the first idea of do no harm. You also have the ability to save the situation.’	Consultation: Recruiter: ‘I have to say it's very difficult. I feel that both treatments would be very good for you. I could sit here and sing the praises of each modality of treatment actually and it's difficult to say which would be best in your situation. And partly, if you're looking for something in the short‐term that was good, then you might say, “Well, HIFU has less side effects up front.” But in the longer term there's uncertainty about whether you need repeat treatment, on‐going monitoring, all of that uncertainty. We don't know what the long‐term results are – 10, 15, 20 years – we don't know the results of focal treatment. Whereas with surgery we know those true outcomes, but we know also that it carries a greater burden of side effects. I can't tell you which of those packages is best overall, only this kind of study will tell us that.’
	Consultation: Patient: ‘I think my concern, I mean I was interested in HIFU because it carries the least possible side effects. I know it's in its infancy but I think‐’ Recruiter: ‘A bit beyond infancy I would say but yes.’ Patient: ‘About 15 years?’ Recruiter: ‘I think HIFU has been probably around for that sort of time. Focal therapy treatment has probably been going from around 2007/8, something like that. We've got quite a lot of outcome which is going to be published. […] We have some confidence that the results will be okay otherwise we wouldn't be doing it.’	Consultation: Patient: ‘HIFU sounds like an attractive idea.’ Recruiter: ‘I think the important thing to realise is that we also think it's an attractive idea…but it's very important in what we do to establish evidence to really know that attractive ideas turn out to be good ideas […] The only problem is that we don't have long term follow up. We certainly don't have this randomised evidence. There is a body of opinion which says that in your case, intermediate‐risk prostate cancer, we don't really know which is better; surgery with whole gland therapy or focal therapy.’
Recruiter 2	Consultation: Recruiter: ‘I can tell you why I think surgery is good and what's great about surgery, but I'm not here to tell you what is great or not great about radiotherapy or HIFU.’	Consultation: Recruiter: ‘I don't know [if HIFU is as good as surgery] because we haven't done the study yet. But the data would support that it appears to be as good, yes. But I can't answer that question in terms of cancer control. What I can say for sure is that if you had the HIFU, you're likely to have a quicker recovery. You'll be out of hospital quickly. Generally people are less tired afterwards, less fatigued and they tend to have a quicker recovery from the HIFU treatment. And it probably has less impact on erections. It certainly has less impact on incontinence.’
	Consultation: Recruiter: ‘I think surgery would be a good treatment choice for you.’	Consultation: Recruiter: ‘In terms of advice […] it's not for me to tell you what treatment to have […] I'm here to tell you what are the pros and cons of different treatments.’

## Discussion

The PART study aimed to evaluate the feasibility of recruiting men with intermediate‐risk prostate cancer to HIFU or RP. A QRI was integrated to identify and address barriers to recruitment. Early in the feasibility study, in Phase I of the QRI, recruiters without previous experience of recruitment found the concept of equipoise difficult and often disclosed their views about the most suitable treatment for patients. In Phase II of the QRI, several strategies were implemented to enable recruiters to discuss their views about the treatments and the trial, and then support them to convey uncertainty and equipoise to patients more clearly. There was an increase in recruitment from 1.4 patients/month in Phase I of the QRI to 4.5 patients/month in Phase II. After the first QRI intervention in December, centres began recruiting consistently. There was also evidence to suggest that the QRI had influenced clinicians’ practices and led to clear presentations of equipoise to patients. Whilst it is not possible to determine the precise impact that the QRI had on recruitment, this suggests it had a positive impact. The PART study randomised a total of 82 men, showing that it is feasible to recruit to an RCT of RP vs HIFU, and paves the way for a definitive and potentially practice‐changing large Phase III RCT, currently in preparation.

Previous research has shown that recruiters can find the dual roles of clinician and researcher conflicting 11. Their experience can lead them to favour one treatment in general or for patients with particular disease characteristics or health states. A recent study of six trials showed that even when recruiters intended to convey equipoise to patients, they often failed to do so or provided unbalanced information, with some undermining equipoise with recommendations 9. A systematic review indicated that didactic‐based learning may not necessarily be most effective for recruitment training 12. In PART, training and support was delivered in a way that encouraged discussion and collaborative decision‐making about equipoise and uncertainty, so that recruiters could find their own position of equipoise and then understand how they could communicate this more clearly to trial patients. The present study indicates that it is possible to change how recruiters present information to patients. Moreover, the confidence of recruiters who received training from ProtecT 13 suggests that the effect of this support may be sustained.

The main strength of the present research was the use of qualitative methods to provide insights into clinician equipoise in real consultations. The QRI adopted a range of qualitative data collection methods to gain an in‐depth understanding of recruitment processes, how the trial was presented, and how patients were responding to the trial. It was also possible to compare what was intended (in interviews) with what was actually expressed in consultations. Thus, interviews showed participants’ intention to be neutral, but the consultations showed ways in which they unintentionally steered patients towards particular treatments. Recorded consultations also enabled us to compare how recruiters with or without previous experience presented equipoise before and then after training. The opportunity to feedback findings quickly to change practices was a key strength, highlighting the applied nature of the QRI 14.

The present study has several limitations. It was conducted in one trial with an observational design, and so findings should be interpreted with caution. It is not possible to evaluate the causal effects of the QRI in this small study, but a formal evaluation of the effectiveness of QRIs implemented to date on the numbers of eligible patients approached and conversion rates will be presented in due course. It is challenging to quantify changes in recruiter communication with patients. Innovative methods have been developed to assess the balance of information provision 15 and provide evidence of participant understanding 16. It will be important in future work to develop ways to quantify the effects of the training and support. In terms of sampling, the views of those who agreed to be interviewed may not be representative of all clinicians (for instance, the site leads may have been more enthusiastic about the study than other clinicians at their centre). However, even amongst these, diverse opinions were expressed. Many research nurses were also unavailable for interviews or did not respond to the QRI researcher. Furthermore, despite QRI encouragement and support, sites did not regularly record all consultations (as has been the case in previous QRIs 14, 17, 18, 19). This sometimes made it difficult to provide tailored and specific feedback, limiting the potential impact the QRI might have had.

In summary, recruiters can find it difficult to enrol patients in a trial comparing very different treatment arms, such as PA and RP. This research suggests that these challenges can be overcome by targeted training and support to enable recruiters to become more comfortable with the concept of uncertainty and then have confidence to approach eligible patients and present equipoise clearly to facilitate informed decision‐making and trial participation.

## Funding

This PART feasibility study was funded by the National Institute for Health Research (NIHR) Health Technology Assessment (HTA) programme (12/35/54). This work was also supported the Medical Research Council (MRC) ConDuCT‐II Hub (Collaboration and innovation for Difficult and Complex randomized controlled Trials In Invasive procedures – MR/K025643/1) and the Royal College of Surgeons of England Bristol Surgical Trials Centre. Jenny L. Donovan was supported in part by the NIHR Collaboration for Leadership in Applied Health Research and Care (CLAHRC) West at University Hospitals Bristol NHS Foundation Trust. Freddie C. Hamdy is supported by the Oxford NIHR BRC Surgical Innovation and Evaluation Theme. HUA receives funding from Wellcome Trust, Prostate Cancer UK and has previously received funding from the NIHR. He also receives funding from Sophiris Inc., Sonacare Inc. and Trod Medical, and is supported by infrastructure support provided by the NIHR Imperial Biomedical Research Centre. Mark Emberton receives funding from UCLH/UCL NIHR Biomedical Research Centre. Jenny L. Donovan, Mark Emberton and Freddie C. Hamdy are NIHR Senior Investigators. This article presents independent research funded by the MRC and NIHR. The views expressed are those of the authors and not necessarily those of the MRC, NHS, NIHR or the Department of Health.

## Conflicts of Interest

None.

**Abbreviations**
HIFU: high‐intensity focused ultrasound
PA: partial ablation
PART: Partial prostate Ablation vs Radical prostaTectomy (study)
ProtecT: Prostate Testing for Cancer and Treatment (trial)
QRI: QuinteT Recruitment Intervention
RCT: randomised controlled trial
RP: radical prostatectomy
TMG: Trial Management Group

## Supporting information


**Table S1.** Consolidated criteria for reporting qualitative research (COREQ).

**Appendix S1.** PART QRI: Topic guide for recruiters (including TMG members). PART QRI: Topic guide for non‐recruiting TMG.

**Appendix S2.** PART QRI: Key issues identified in tips document.
