# Supplementary material for: Overcoming difficulties with equipoise to enable recruitment to a randomised controlled trial of partial ablation vs radical prostatectomy for unilateral localised prostate cancer
Source: BJU Int. 2018 Aug 15;122(6):970–7. doi: 10.1111/bju.14432 (PMC6348419; doi:10.1111/bju.14432)
Supplement: Supplementary file 1 — Table S1. Consolidated criteria for reporting qualitative research (COREQ). [file BJU-122-970-s001.docx]

Table 1: Consolidated criteria for reporting qualitative research (COREQ)

|  | **No** | **Item** | **Guide questions/description** | **Comment** |
| --- | --- | --- | --- | --- |
| **Domain 1: Research team and reflexivity** | | | | |
| **Personal characteristics** | 1 | Interviewer/facilitator | Which author/s conducted the interview or focus group? | DE conducted 11 interviews, PW conducted 2 interviews |
|  | 2 | Credentials | What were the researcher’s credentials? e*.g. PhD, MD* | DE – BSc and PhD in Psychology  PW – BSc, MA, PhD in Sociology |
|  | 3 | Occupation | What was their occupation at the time of the study? | DE – Senior Research Associate in Health Services Research  PW - Senior Research Associate in Health Services Research |
|  | 4 | Gender | Was the researcher male or female? | DE – female, PW - male |
|  | 5 | Experience and training | What experience or training did the researcher have? | Both DE and PW have several years of experience conducting qualitative research. This has included conducting and completing qualitative projects and attending training courses and workshops. |
| **Relationship with participants** | 6 | Relationship established | Was a relationship established prior to study commencement? | No |
|  | 7 | Participant knowledge of the interviewer | What did the participants know about the researcher? *e.g. personal goals, reasons for doing the research* | The researchers introduced themselves, explained the purpose of the research and provided an information leaflet about the study |
|  | 8 | Interviewer characteristics | What characteristics were reported about the interviewer/facilitator? *e.g. Bias, assumptions, reasons and interests in the research topic* | The researchers explained how the QRI related to the PART trial |
| **Domain 2: study design** | | | | |
| **Theoretical framework** | 9 | Methodological orientation  and theory | What methodological orientation was stated to underpin the study? *e.g. grounded theory, discourse analysis, ethnography, phenomenology, content analysis* | Data were analysed thematically using techniques of constant comparison derived from grounded theory methodology |
| **Participant selection** | 10 | Sampling | How were participants selected? *e.g. purposive, convenience, consecutive, snowball* | Purposeful and snowball:  Initial, scene-setting interviews were conducted with members of the Trial Management Group (TMG) (including the Chief Investigator (CI)). Snowball sampling was subsequently used, where TMG members provided the names of colleagues who were considered beneficial for the QRI researcher to talk to.  Informants were purposefully selected to capture a range of perspectives (in terms of varying individual recruitment rates, ensuring there was at least one representative from each site, and with speaking to those with varying expertise/views of radical prostatectomy or HIFU). Characteristics were assessed as the study progressed, and some individuals were subsequently selected on the basis of emerging issues that warranted further investigation (i.e. the evidence for HIFU) or were approached as new centres opened throughout the course of the study. |
|  | 11 | Method of approach | How were participants approached? e*.g. face-to-face, telephone, mail, email* | Healthcare professionals were contacted by the researcher via email. Patients were approached face to face by healthcare professionals. |
|  | 12 | Sample size | How many participants were in the study? | A total of 13 one-to-one interviews were conducted. Sixty-four recruitment appointments with 54 patients were audio-recorded (five patients had two consultations recorded) |
|  | 13 | Non-participation | How many people refused to participate or dropped out? Reasons? | The QRI researcher approached 23 healthcare professionals to take part, and 13 of these took part in an interview. However, not all PART consultations were recorded and there was considerable variability in how many recordings each site provided. Healthcare professionals frequently cited lack of time or logistical issues. |
| **Setting** | 14 | Setting of data collection | Where was the data collected? *e.g. home, clinic, workplace* | Participants chose a location that was convenient for them (their workplace or a nearby café) or opted to do the interview over the telephone. |
|  | 15 | Presence of non-participants | Was anyone else present besides the participants and researchers? | The partners of patients sometimes sat with the patients but spoke very little. |
|  | 16 | Description of sample | What are the important characteristics of the sample? *e.g. demographic data, date* | Key information is provided in the results section. |
| **Data collection** | 17 | Interview guide | Were questions, prompts, guides provided by the authors? Was it pilot tested? | Separate topic guides were developed for the TMG and recruiters (see Additional File) to ensure coverage of overall study issues (TMG) and recruitment (recruiters), with sufficient flexibility to allow for new issues to emerge. An example is included in the supplementary file. |
|  | 18 | Repeat interviews | Were repeat interviews carried out? *If yes, how many?* | No repeat interviews were carried out |
|  | 19 | Audio/visual recording | Did the research use audio or visual recording to collect the data? | Interviews were audio-recorded |
|  | 20 | Field notes | Were field notes made during and/or after the interview or focus group? | The researchers kept notes throughout data collection and analysis |
|  | 21 | Duration | What was the duration of the interviews or focus group? | Interviews lasted an average of 43 minutes (range=31-53 minutes). Consultations lasted an average of 27 minutes (range=10-42 minutes). |
|  | 22 | Data saturation | Was data saturation discussed? | Yes, data collection continued until the team were confident that saturation had been reached. |
|  | 23 | Transcripts returned | Were transcripts returned to participants for comment and/or correction? | Transcripts were not returned to participants for comments or corrections |
| **Domain 3: analysis and findings** | | | | |
| **Data analysis** | 24 | Number of data coders | How many data coders coded the data? | All data were coded by DE. Emerging themes were discussed with JLD with reference to the raw data. |
|  | 25 | Description of the coding tree | Did authors provide a description of the coding tree? | A description of the coding tree is not provided in the article |
|  | 26 | Derivation of themes | Were themes identified in advance or derived from the data? | Themes were derived from the data |
|  | 27 | Software | What software, if applicable, was used to manage the data? | NVivo (version 10) was used to analyse the data |
|  | 28 | Participant checking | Did participants provide feedback on the findings? | Full results were not sent out to all participants to gain respondent validation. |
| **Reporting** | 29 | Quotations presented | Were participant quotations presented to illustrate the themes / findings? *Was each quotation identified? e.g. participant number* | The interpretation of each category is supported by illustrative quotes |
|  | 30 | Data and findings consistent | Was there consistency between the data presented and the findings? | There is consistency between the data presented and the measures developed. |
|  | 31 | Clarity of major themes | Were major themes clearly presented in the findings? | The themes are clearly presented in the findings |
|  | 32 | Clarity of minor themes | Is there a description of diverse cases or discussion of minor themes? | Description of diverse cases and where minor themes occurred between participant groups are discussed (e.g. ProtecT recruiters versus those new to recruiting) |
