# Supplementary material for: Overcoming difficulties with equipoise to enable recruitment to a randomised controlled trial of partial ablation vs radical prostatectomy for unilateral localised prostate cancer
Source: BJU Int. 2018 Aug 15;122(6):970–7. doi: 10.1111/bju.14432 (PMC6348419; doi:10.1111/bju.14432)
Supplement: Supplementary file 2 — Appendix S1. PART QRI: Topic guide for recruiters (including TMG members). PART QRI: Topic guide for non‐recruiting TMG. [file BJU-122-970-s002.docx]

**PART QRI: Topic Guide for recruiters (including TMG members)**

**Opening**

QRI – understand if main trial is possible. Want to understand your views about how the trial is designed and how patients are responding to the trial. Not a medical professional. All completely confidential. Written consent.

1. **Background**
   - How long have you recruited patients to RCTs?
   - How did you become a recruiter?
   - Have you received any training as a recruiter or for RCTs in general?
   - When did you first get involved in this RCT?
   - What is your role in this RCT? Probe for details.
   - Would you describe yourself as a scientist, clinician, doctor/nurse, trialist, researcher, or..? Or do you have more than one role? [If more than one role – are these roles complementary or are there conflicts?]
   - Why do you think this RCT is needed?
   - What is involved in taking part in this RCT; and in each arm?
   - When did recruitment start?
   - How well is recruitment going? Probe for details.
2. **Protocol**
   - Could you describe the key aspects of the protocol?
   - What is the evidence base for this trial? Probe for details of systematic reviews.
   - Are the eligibility criteria for the RCT clear? Do you think PIs agree with them (and comply with them)?
   - Are there reasons why recruitment is/would be difficult in this RCT?
   - Would you say that you are ‘in equipoise’? What does ‘equipoise’ mean to you?
   - Do you think your colleagues (PIs and recruiters) are “in equipoise”? Probe for details.
   - How easy it is for you to accept uncertainty in relation to evidence? How easy is it for PIs/recruiters?
3. **Recruitment pathway**
   - How are patients recruited into this RCT? Is there a “recruitment” appointment?

- Who undertakes the assessment of patient eligibility for this RCT? Are you involved in eligibility assessment?
- How do you find out which patients to approach about the RCT?

1. **Introducing/explaining the trial**- How do you introduce/explain the trial to patients?
   - Why do you think this RCT is needed?
   - What is involved in taking part in this RCT; and in each arm?
   - What happens to patients if they agree to take part?
2. **Randomisation**
   - How do you explain to patients how the decision is made in the trial about which treatment they
   will receive?
   - How easy do you think the concept of randomisation is for patients to understand?
   ***Note****: If this seems threatening, acknowledge that this is a difficult and confusing concept for everybody
   and then explore again.*- Does randomisation seem sensible and reasonable to you?
3. **Uncertainty**
   - How do patients react to the idea that the specialist doctor does not know what the best treatment is?
   - How do you describe the uncertainty?
   - How do you feel about explaining uncertainty?
   - Do you ever have a feeling during an appointment that a patient should really have one treatment rather than another? Probe: Why? What do you do about that?
4. **Informed consent**- How easy do you think it is to obtain fully informed consent for patients participating in this RCT?
   (Probe: what is informed consent; how do you know when you have reached it?)
   - Are you ever concerned about the possibility of coercing patients into taking part in the trial?
5. **Patients**- How do you think patients feel about taking part in RCTs in general?
   - How do you think these patients feel about taking part in this RCT?
   - Do patients express preferences for particular treatments?
   (Probe – what are their preferences; do you know why they express preferences?)
   - What do you do when a patient expresses a preference?
   (Probe – do you accept it – what happens next? Or do you ever explore or challenge their preference?)
   - What other reasons (not preferences) have patients given for not taking part in this RCT?
   - What do you do when a patient gives a reason for not wanting to take part?
6. **Difficulties during recruitment**
   - What would you say are the main difficulties you face as a recruiter?
   - Can you describe specific examples of ‘good’ and ‘bad’ recruitment experience***s***- Is recruitment organised well? Do other people explain the RCT to patients?
   - Are there difficulties with any particular arm?
   - Do patients have strong preferences?
   - Do you think this RCT is the right thing for these patients**?**
7. **Personal views**- Do you think your work as a recruiter is similar or different from your usual work/practice? Probe: In what ways it is (a) similar, (b) different?
   - Do you think your work as a recruiter has changed the way you practice outside research?
   - How do you feel about being a recruiter? Probe: Do you see yourself as a doctor/nurse/clinician, scientist, or researcher (if nurse also add ‘patient advocate’); or all of these? Are there benefits or problems that arise from these different roles?
   - Do you think this RCT will be successful? Probe all the reasons for answer.
   - Do you know (or have a hunch) about what the outcome of the RCT will be?
   - If you were a patient in this position today, would you agree to be recruited to the RCT and be randomised? Or would you choose a treatment? Which one?
8. **Finally - improving recruitment**
   - What might, in your opinion, improve the recruitment appointments?
   - Do you think you (or others) need more support or training?

   **Closing**

Summarise key points, check if there are any further questions, discuss logistics of recording consultations in centres and thank for taking part.

**PART QRI: Topic Guide for Non-Recruiting TMG**

**Opening**

QRI – understand if main trial is possible. Want to understand your views about how the trial is designed and how patients are responding to the trial. Not a medical professional. All completely confidential. Written consent.

**Background**

- Can you tell me about your role in PART*? (Probe: Recruiting, identifying eligible patients, recruiting eligible patients, protocol development?)*
- Could you tell me about how and why you got involved in PART?
- What centres are involved? *(Probe: How were they selected? Thoughts on how they will do? Any particular challenges?)*
- What is recruitment target? How recruiting going so far?

**Recruitment in PART**

- Could you explain, in your own words, what the PART study is about? What is involved in taking part in PART; and in each arm?
- Could you tell me about the different treatment options outside of the trial that are available for men with intermediate risk, unilateral clinically localised prostate cancer? (*Probe: Active monitoring? Radiotherapy? Brachytherapy?)*
- Do you think there’s a need for this trial? (*Probe: Reasons for this)*
- Can you talk me through previous research done in this area? Index Lite? Index? ProtecT?
- How do you think recruitment is going so far? Do you think PART will be successful?

**Eligibility criteria**

- Could you describe the eligibility criteria to me? (*Probe for thoughts on the criteria…particularly limits of eligibility criteria)*.
  - Can you tell me what MRI/biopsies are needed to determine eligibility? When are they performed? PROMIS trial? (Conventional TRUS biopsy verses mpMRI). Will findings change practice, will they affect PART?
  - Gleason score? (4+3, or 3+4)
  - Unilateral disease?
  - PSA?
  - Life expectancy/age?
- Do you think recruiters will use any other criteria (e.g. non-clinical)? Any concerns?

**The pathway**

- Can you talk me through what the patient pathway is like? Differences in any centres?

**Discussing the trial**

- How do you think recruiters will introduce/explain the PART study to patients? Fit with other treatment options?
- How do you think patients will respond to the trial? Preferences? Reasons for declining?
- Any challenges with a particular arm?

**Recruiter hunches and preferences**

- Do you think the feasibility study will show it’s possible to conduct a full study?
- Do you know (or have a hunch) about what the outcome of PART will be? What do you think about outcomes for this trial?
- If you were a patient in this position today, would you agree to be recruited to the PART study and be randomised? Or would you choose a treatment? Which one?
- What are your thoughts on the design of the PART study?
- How would you design the trial, what arms be?
- What are your thoughts about follow up for this study?

**Closing**

- Summarise key points, check if there are any further questions and thank for taking part.
