# Supplementary material for: Overcoming difficulties with equipoise to enable recruitment to a randomised controlled trial of partial ablation vs radical prostatectomy for unilateral localised prostate cancer
Source: BJU Int. 2018 Aug 15;122(6):970–7. doi: 10.1111/bju.14432 (PMC6348419; doi:10.1111/bju.14432)
Supplement: Supplementary file 3 — Appendix S2. PART QRI: Key issues identified in tips document. [file BJU-122-970-s003.docx]

**PART QRI: Key issues identified in tips document**

Recruiters were encouraged to:

- Approach all patients about the PART study if they had intermediate risk localised prostate cancer
- Reassure the patient that prostate cancer is a very common disease in men
- Establish uncertainty at the beginning of the consultation
- Mention the study early on
- Explain the benefits of study participation
- Provide an overview of the treatment options (active surveillance/radical treatment/partial treatment)
- Present balanced information about both HIFU and surgery
- Gently find out the reasons why a patient prefers one option over the other
- Avoid using terms such as ‘toss of a coin ‘or ‘decided by a computer’ to explain randomisation
- Explain the rationale for randomisation
